# Recreational Marijuana Use and Coronary Artery Dissection: A Case Series

**DOI:** 10.7759/cureus.21778

**Published:** 2022-01-31

**Authors:** Adeyinka Adeniyi, Sandra Abadir, Mohammed Kooshkabadi, Sunday O Yusuf, Radhika Khanna, Biagio Collura, Marie Anais Hichard

**Affiliations:** 1 Internal Medicine, Wellstar Atlanta Medical Center, Atlanta, USA; 2 Cardiology, Wellstar Atlanta Medical Center, Atlanta, USA; 3 Family Medicine, Wellstar Atlanta Medical Center, Atlanta, USA

**Keywords:** coronary artery spasm, acute coronary syndrome, illicit drugs, coronary artery dissection, scad, marijuana

## Abstract

The cardiovascular effects of marijuana have been shown to be a result of the activation of the CB1 and CB2 receptors located in the myocardium and coronary vasculatures. Adverse cardiovascular consequences of recreational use of marijuana and synthetic cannabinoids include stroke, artery dissection, vasospasm, vasculitis, coronary artery thrombosis, myocarditis/pericarditis, postural hypotension, arrhythmias, and acute heart failure. With marijuana being legalized for medicinal purposes and recreational use in more and more states in the United States, physicians should have a low threshold for the possibility of marijuana being the underlying cause of adverse cardiovascular events. Marijuana has been established to increase sympathetic tone and cause blood pressure elevations and severe coronary artery spasm (CAS). Some studies have even indicated that the risks of heart attack, atrial fibrillation, and ischemic stroke are several times higher within an hour of marijuana use. This case series discusses three female patients, aged 27, 39, and 53 years, who were known to smoke marijuana consistently. These patients all presented with signs and symptoms of acute coronary syndrome (ACS) less than 12 hours after smoking recreational marijuana. All patients endorsed smoking marijuana a few hours prior to the onset of chest pain and less than 12 hours prior to the presentation, which was confirmed by a positive urine drug screen (UDS). Coronary artery angiograms revealed coronary artery dissection in the proximal left circumflex (LCX) artery, the mid-distal left anterior descending (LAD) artery, and mid-LAD in the 27 y/o, 39 y/o, and 53 y/o patients respectively. The average age of spontaneous coronary artery dissection (SCAD) cases ranges between 35-40 years. Women account for more than 70% of cases; it is thought to be due to higher levels of estrogen in women, which alters the normal arterial wall architecture. Additionally, the excessive use of marijuana resulting in CAS further increases the susceptibility to spontaneous dissection in female patients.

## Introduction

Spontaneous coronary artery dissection (SCAD) is a type of coronary artery dissection affecting young healthy adults, which is not atherosclerotic, traumatic, or iatrogenic [1]. It was first discussed in the context of an autopsy of a 42-year-old female in 1931 [2]; since then, many cases of SCAD have been reported, contributing to the evolution of our understanding of this condition. Clinical presentation of SCAD depends on the extent and severity of the dissection. SCAD is a diagnosis of exclusion; all other causes of coronary dissection need to be ruled out before it is classified as spontaneous. Myocardial injury in SCAD occurs due to coronary artery obstruction from intimal disruption or intramural hematoma formation [1]. The condition was previously believed to be a rare fatal cause of myocardial infarction (MI) and sudden cardiac death; however, with better means of diagnosis and increased awareness among physicians, SCAD has been found to be more common than we once believed [1]. Women account for 70-80% [2,3] of SCAD cases and most commonly (80%) have the involvement of the left anterior descending (LAD) artery, leading to high mortality rates [3]. The right coronary artery (RCA) is reportedly affected in approximately 20% of cases, and this was seen mostly in men [4].

The presentation of SCAD is dependent on the extent and severity of the dissection. Clinical presentations can range from unstable angina to sudden cardiac death [1]. Although there is no consensus on the optimal treatment for this condition, it is commonly acknowledged that thrombolytics and coronary intervention are not advised as they may expand the dissection [2]. The condition has been highly associated with pregnancy due to pregnancy-related changes in the level of sex hormones and hemodynamics. Increased levels of estrogen induce microscopic changes to the normal arterial wall architecture, leading to increased susceptibility to spontaneous dissection. Hemodynamic changes during pregnancy, such as increased cardiac output and straining during labor, also contribute to the increased arterial wall stress [2]. Other conditions associated with SCAD are physical and emotional stressors, systemic arteriopathies, vasculitis, prolonged sneezing, cocaine abuse, and connective tissue diseases like Marfan syndrome and Ehlers-Danlos type IV [1,2]. Numerous studies and case reports have alluded to the increased risk of SCAD with marijuana use [5]. The purpose of this case series is to further demonstrate the association between marijuana use and the SCAD disease process.

## Case presentation

Case 1

A 27-year-old African American female, gravida 3 para 3 (G3P3), with a past medical history (PMHx) of pulmonary embolism two weeks after her third cesarean delivery, which had been three weeks prior to the admission, presented to the emergency department (ED) with intermittent, sharp, substernal chest pain that radiated to the back. The pain was associated with shortness of breath (SOB) and was exacerbated by exertion. It was rated as 10/10 in severity and was relieved by oxycodone and ibuprofen. The patient denied any fever, nausea, vomiting, diarrhea, abdominal pain, leg swelling, or recent travel. Besides smoking tobacco regularly, she reported intermittent use of marijuana one to two times every other day for seven years.

**Figure 1:**
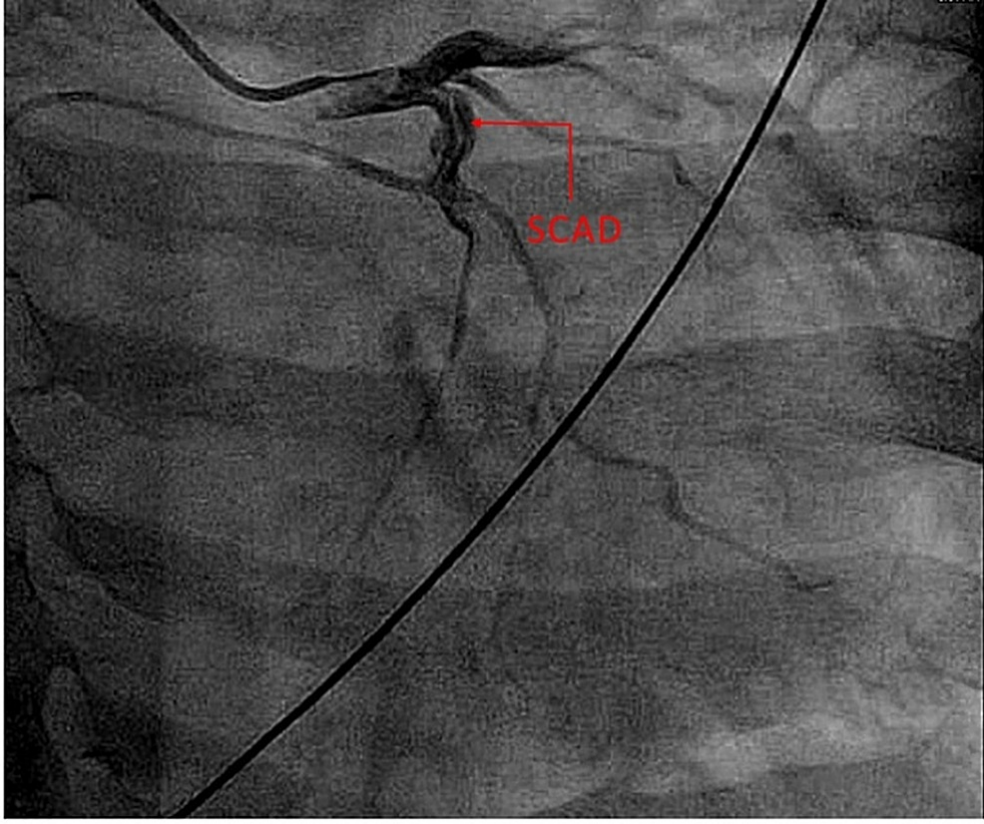
A snapshot of the patient’s coronary angiogram with the red arrow pointing to the area of dissection at the proximal left circumflex artery SCAD: spontaneous coronary artery dissection

Her vitals were within the normal limits and physical examination revealed decreased chest wall expansion, coarse rhonchi, and prolonged respiratory phase. Laboratory findings were significant for troponin I of 0.64 ng/ml x1 (peaked to 1.87 ng/mL x2, 4.65 ng/ml x3). Urine toxicology was positive for tetrahydrocannabinol (THC). Electrocardiography (ECG), chest X-ray (CXR), and transthoracic echocardiogram (TTE) were unremarkable. Coronary angiography revealed evidence of spontaneous coronary artery dissection in the proximal left circumflex (LCX) artery with Thrombolysis in Myocardial Infarction (TIMI) grade III flow, as shown below in Figure 1 and Video 1.

The patient was started on dual antiplatelet therapy, with clopidogrel and rivaroxaban, to be continued for one year, and was advised to avoid strenuous activity so that the dissection would heal. She was encouraged to avoid marijuana use and to follow up two months after discharge for re-imaging.

**Video 1:** Coronary angiogram pointing to the area of dissection at the proximal left circumflex artery

Case 2

A 39-year-old African American female, with PMHx of pre-eclampsia and hyperlipidemia managed with diet, presented to the ED via ambulance complaining of a sudden onset of "squeezing" chest pain that radiated to the left arm. The pain had started suddenly while she had been driving, and it was rated as 8/10 in intensity. The pain lasted for 20-30 minutes and was associated with diaphoresis and SOB. Her pain resolved spontaneously and the left arm numbness improved after she took an aspirin in the ED. She noted that she had been having persistent left shoulder, left neck, and left-back pain for three days prior to the acute episode, which she attributed to sleeping in a wrong position. She denied cough, fever, chills, nausea, vomiting, or any other symptoms. The patient denied the use of tobacco but reported smoking marijuana every other day for over 20 years.

**Figure 2:**
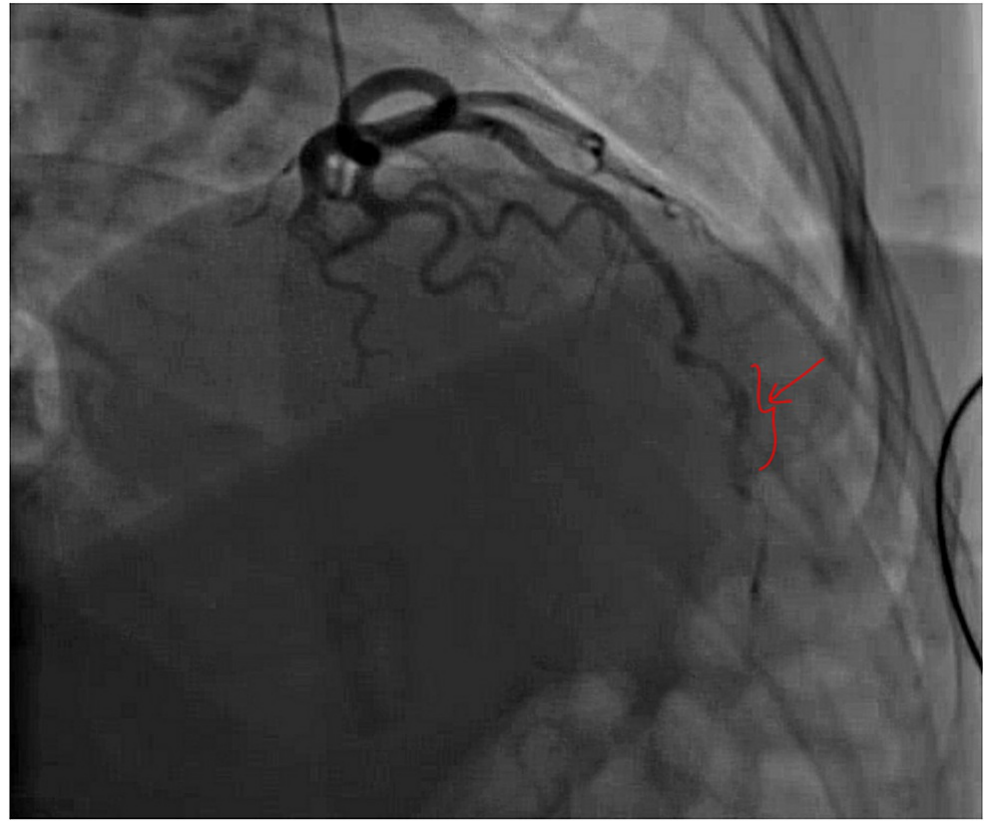
A snapshot of the patient’s coronary angiogram with the red arrow pointing to the area of dissection in the mid-distal LAD artery LAD: left anterior descending

Her vital signs and physical examination were unremarkable; laboratory findings were significant for troponin I of 0.10 ng/ml x1 (peaked to 1.36 ng/ml x2), and urine toxicology was positive for THC. Her ECG, CXR, and TTE were normal and coronary angiography showed evidence of mid-distal LAD SCAD with TIMI grade II flow, as seen in Figure 2.

Cardiothoracic surgery was consulted and no surgical intervention was required; she was started on the acute coronary syndrome (ACS) protocol and medically managed with a heparin drip, carvedilol, aspirin, atorvastatin, clopidogrel, hydralazine, and isosorbide mononitrate. She was advised to quit marijuana and follow up with a cardiologist in two months.

Case 3

A 53-year-old female with PMHx of asthma, thyroid cancer status post thyroidectomy, acquired hypothyroidism, and acid reflux presented to the ED with cramping substernal chest pain that radiated to the left arm. The pain was associated with weakness, nausea, vomiting, and diaphoresis and was described as 10/10 in severity. She denied any tobacco use but reported chronic use of marijuana with the last use occurring less than 12 hours before the presentation.

**Figure 3:**
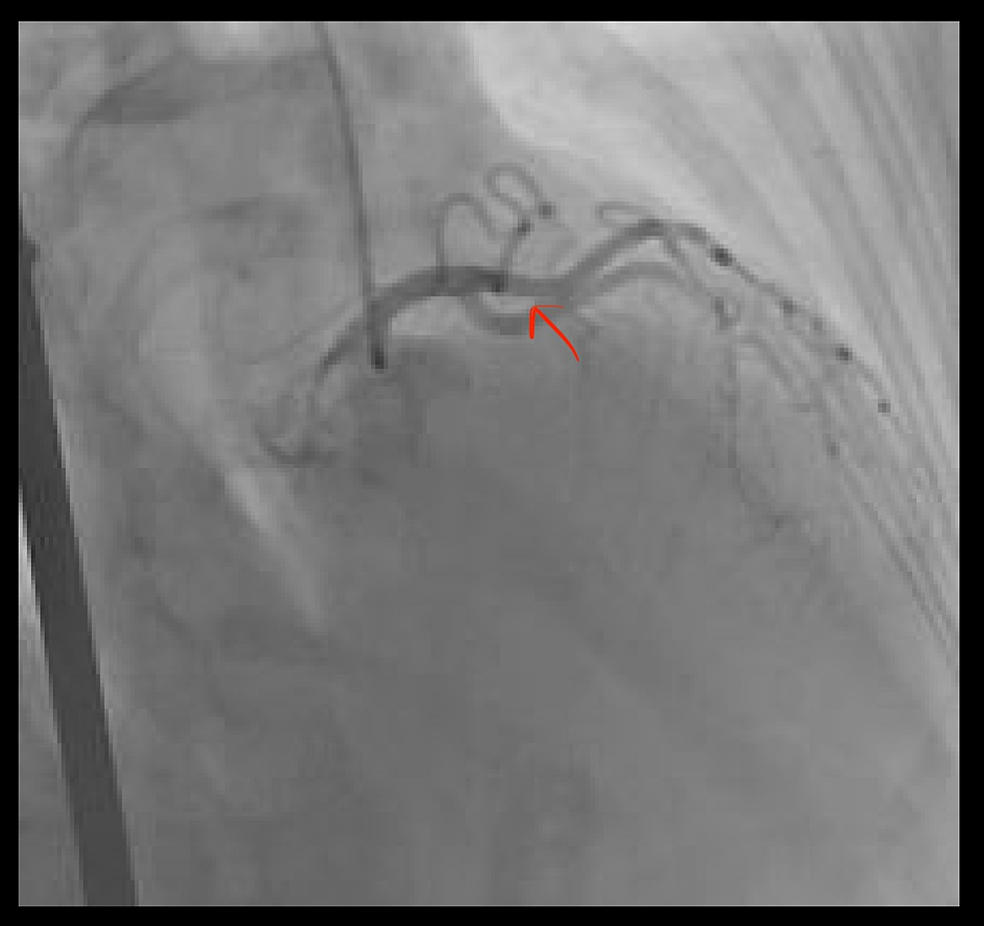
A snapshot of the patient’s coronary angiogram with the red arrow pointing to the area of dissection in the mid-LAD artery LAD: left anterior descending

Her vital signs and physical examination were unremarkable. CXR was normal and ECG revealed ST elevation in leads V2 and V3. Her laboratory values were significant for troponin I of 0.10 ng/ml x1 (peaked to 0.58 ng/ml x2). Urine toxicology was positive for THC. A left heart catheterization revealed coronary artery dissection in mid-LAD with small filling defects seen at distal LAD (Figure 3) with TIMI III flow; coronary whisper wire was unable to be cross-lesioned for the purpose of intravascular ultrasound (IVUS), and hence the plan was aborted. 

The patient was managed with aspirin, heparin drip, nitroglycerin, and nicardipine drip. Due to persistent severe chest pain, antiplatelets were not given and she was transferred to a facility capable of performing a coronary artery bypass graft (CABG) surgery.

## Discussion

SCAD is an underdiagnosed condition causing severe chest pain in young previously healthy individuals, mainly women [1]. The condition is misdiagnosed due to factors such as physician unfamiliarity, limitations of coronary angiographic techniques, and low suspicion of ACS in younger women [1]. Patients’ presentation differs based on the extent and severity of the dissection, but most patients present with ACS symptoms and elevated cardiac enzymes [1].

Early catheterization and conventional coronary angiography are necessary to establish a diagnosis and guide further management [3]. Catheterization showing a radiolucent line dividing true and false lumen of the coronary artery is diagnostic for SCAD [3]. Angiography suggestive of SCAD reveals multiple radiolucent lumens with extraluminal contrast leaking [1]. Unfortunately, these findings are seen only in a segment of SCAD patients [1] while some present with only luminal narrowing, which is misinterpreted as arthrosclerotic plaque [6]. The use of IVUS and optical coherence tomography further helps locate the exact location of the dissection and its extent; however, they can also worsen the presenting dissection [4].

The exact mechanism behind cannabis-induced SCAD is not fully understood, but it is believed to be due to a combination of increased sympathetic drive causing an increase in shear stress on the walls of the coronary arteries and thereby leading to SCAD. As described by Kariyanna et.al and Filali et al. [7,8], THC activates cannabinoid (CB1 and CB2) receptors found in multiple tissues. In the heart and blood vessels, this activation causes sympathetic stimulation in a dose-dependent fashion and increases heart rate, peripheral vasodilation, systolic and diastolic blood pressures, and cardiac output. Additionally, THC increases cardiac arrhythmias and ACS risk within an hour of smoking marijuana. Carboxyhemoglobin is seen to rise after cannabis smoking and this reduces oxygen-carrying capacity. This increases the oxygen demand while reducing the blood supply, leading to myocardial ischemia. Furthermore, cannabis smoking causes plaque disruption by intensifying oxidative stress, increasing the formation of oxidized low-density lipoprotein, and enhancing the activity of factor VII and platelets. The plaque disruption along with increased coagulation precipitates thrombosis. 

Due to the lack of management guidelines, protocols used for ACS management are used in the management of SCAD. Conservative management with medication in hemodynamically stable patients has shown benign hospital course and angiographic coronary dissection resolution within a year [2]. The use of dual antiplatelet therapy, heparin, and beta-blockers is reported to keep the patency of the true lumen, prevent thrombosis, and speed up healing; however, thrombolytics should be avoided as they increase bleeding and intramural hematoma [4]. The first line of management in SCAD patients with acute MI with evidence of hemodynamic instability or ischemia, complete vessel occlusion, recurrent chest pain, and sustained ventricular arrhythmias remains rapid revascularization with percutaneous coronary intervention (PCI) or CABG to establish coronary blood flow [4]. PCI has been shown to be associated with higher rates of failure in passing wires into the false lumen of the dissected coronary artery, and loss of blood flow has been reported [6]. These challenges limit the use of PCI in individuals presenting with ongoing ischemia, and the utilization of real-time IVUS during the procedure is encouraged [6].

The increasing consumption of cannabis for medical reasons and for recreation calls for further investigations into the adverse effects caused by acute and chronic use of the substance. Some cardiovascular conditions known to be caused by marijuana include cardiac arrhythmias, sudden cardiac death, increased systolic blood pressure, and MI [5]. The three patients presented here were otherwise healthy individuals who experienced SCAD after the consumption of marijuana. Although definitive research about the cardiovascular effects of marijuana has been scarce, it has been established to increase sympathetic tone and cause acute coronary artery spasm, which predispose patients to coronary artery pathology [5].

## Conclusions

The use of marijuana contributes to multiple cardiovascular pathologies including arrhythmias, stroke, increased blood pressure, MI, sudden cardiac death, and the progression of SCAD. Young healthy females presenting with ACS symptoms should be queried about the use of cannabinoids; SCAD should be suspected in these patients. Moreover, patients with risk factors for SCAD, including peripartum or postpartum female patients, should be advised and counseled accordingly about the potential risk of developing SCAD and the contributory effect of marijuana in the disease process.
